# Reply to Demiral et al. Comment on “Mahmood, M.N. Direct Immunofluorescence of Skin and Oral Mucosa: Guidelines for Selecting the Optimum Biopsy Site. *Dermatopathology* 2024, *11*, 52–61”

**DOI:** 10.3390/dermatopathology12030027

**Published:** 2025-09-04

**Authors:** Muhammad N. Mahmood

**Affiliations:** Department of Laboratory Medicine and Pathology, University of Alberta Hospital, Edmonton, AB T6G 2B7, Canada; mmahmood@ualberta.ca; Tel.: +1-780-407-2145

I have reviewed the insightful comments on my review article titled “Direct Immunofluorescence of Skin and Oral Mucosa: Guidelines for Selecting the Optimum Biopsy Site” [1]. I sincerely appreciate the interest shown in my work and value the thoughtful and constructive feedback that has been provided. I welcome the opportunity to elaborate on the points raised and to contribute further to the academic discourse on this topic.

As the title of my article states, the purpose of my review was to provide comprehensive guidelines for selecting the optimum biopsy site for direct immunofluorescence (DIF) in skin and oral mucosa. The article is not an attempt to provide a general review of DIF findings in autoimmune vesiculobullous diseases, vasculitis, and connective tissue disorders. This information is already accessible to students of the field through many previously published reviews and texts [2,3]. My article delivers a focused and nuanced evidence-based approach for selecting the appropriate biopsy site, targeting dermatology residents as the primary audience. In their commentary, Demiral et al. provide a general summary of various diseases for which DIF studies are utilized [4]. They acknowledge that my compilation is educational and serves as a valuable resource for clinicians; however, their main text does not provide a direct critique or discuss the specific points highlighted in my review. While their commentary offers a very concise, mainly clinical overview of various cutaneous conditions requiring DIF, it is strictly not a commentary on my review.

A picture is worth a thousand words, and clinical images of dermatological conditions can be an invaluable resource [5,6]. The table with clinical pictures provided in the comment by Demiral et al. is both exciting and a welcome addition. Table 1 in my review summarizes key recommendations; however, the images added by the authors provide an added visual dimension. Marking the exact site to biopsy on a clinical image helps clinicians better understand the points discussed in my review. This visual representation of the concept can serve as a quick reference for clinicians.

My only critique is that the table contains too many images, making it appear busy and cluttered. Creating two or three tables that contain related conditions could be more visually appealing and easier to comprehend for readers. Another option is to include links to Table 1 in my review, which direct readers to relevant images. Any trainee in the field can utilize the information from my review and create their visual cheat sheet. It can be a worthwhile educational exercise and can assist them when performing biopsies for DIF. However, my critique does not detract from the idea of adding a visual dimension to enhance the understanding of the concepts discussed in my review [1].

In conclusion, the inclusion of a table with images in the comment is a valuable addition that enhances the guidelines for selecting the best biopsy site for DIF. I want to thank the authors once again for their active engagement with my work. Exchanges like these are essential for the ongoing advancement of clinical practice and patient outcomes.

## Abbreviations

The following abbreviation is used in this manuscript:

DIF	Direct Immunofluorescence

## References

[1] Mahmood M.N. (2024). Direct Immunofluorescence of Skin and Oral Mucosa: Guidelines for Selecting the Optimum Biopsy Site. Dermatopathology.

[2] Kim R.H., Brinster N.K. (2020). Practical Direct Immunofluorescence. Am. J. Dermatopathol..

[3] Ghanadan A., Saghazadeh A., Daneshpazhooh M., Rezaei N. (2015). Direct immunofluorescence for immunobullous and other skin diseases. Expert. Rev. Clin. Immunol..

[4] Demiral Ş., Özcan Y., Gamsızkan M. (2025). Comment on Mahmood, M.N. Direct Immunofluorescence of Skin and Oral Mucosa: Guidelines for Selecting the Optimum Biopsy Site. *Dermatopathology* 2024, *11*, 52–61. Dermatopathology.

[5] Mahmood M.N. (2021). A Picture Says a Thousand Words. The Importance of Digital Clinical Photography in Establishing Histopathological Diagnosis of Cutaneous Disorders: A Dermatopathologist’s Perspective. J. Prim. Care Community Health.

[6] Cerroni L., Argenyi Z., Cerio R., Facchetti F., Kittler H., Kutzner H., Requena L., Sangueza O.P., Smoller B., Wechsler J. (2010). Influence of evaluation of clinical pictures on the histopathologic diagnosis of inflammatory skin disorders. J. Am. Acad. Dermatol..

